# Gold-catalyzed oxidation of arylallenes: Synthesis of quinoxalines and benzimidazoles

**DOI:** 10.3762/bjoc.7.98

**Published:** 2011-06-24

**Authors:** Dong-Mei Cui, Dan-Wen Zhuang, Ying Chen, Chen Zhang

**Affiliations:** 1 College of Pharmaceutical Science, Zhejiang University of Technology, Hangzhou 310014, PR China; 2 College of Pharmaceutical Sciences, Zhejiang University, Hangzhou 310058, PR China

**Keywords:** allene, benzimidazole, gold-catalysis, oxidation, quinoxaline

## Abstract

A gold-catalyzed oxidation of arylallenes to form α-diketones and aldehydes in good yields is presented. Further directed synthesis of quinoxalines and benzimidazoles, via the condensation of the resulting α-diketones and aldehydes with benzene-1,2-diamine, was achieved in high yields.

## Introduction

Recently, several research groups have developed gold-catalyzed homogeneous catalytic reactions [1]. A variety of organic transformations have been shown to be mediated by gold(I) or gold(III) complexes in solution. In addition to its ability to activate unsaturated C–C bonds, the catalysis of nucleophilic addition by gold complexes for the formation of carbon–carbon and carbon–heteroatom bonds has been one of the most investigated reactions in recent organometallic catalysis [1–24]. In particular, water as a nucleophilic reagent has been used in the addition of alkynes and allenes [16–18]. In contrast, gold-catalyzed oxidation chemistry has been less well developed [25–36], although oxidative cleavage of carbon–carbon double bonds and carbon–carbon triple bonds by homogeneous gold catalysts was reported recently [28–29,33]. To the best of our knowledge, gold and other transition metal-catalyzed oxidations of allenes have not been reported [37–38]. In the context of ongoing studies on metal-catalyzed atom-economical reactions, we have been interested in the use of gold for simple and highly efficient transformations. Additionally, quinoxaline and benzimidazole skeletons are common building blocks for the preparation of substances with pronounced biological activities [39–44]. Herein, we report the gold(I)-catalyzed oxidation/hydration and oxidative cleavage of allenes to form α-diketones and aldehydes, and the synthesis of quinoxalines and benzimidazoles via the condensation of the resulting α-diketones and aldehydes with benzene-1,2-diamine [45–56].

## Results and Discussion

**Scheme 1:**
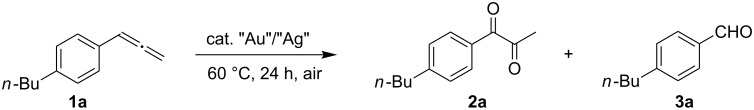
Oxidation of 4-butylphenylallene.

Our initial explorations focused on the reaction of 4-butylphenylallene (**1a**) (0.5 mmol) in the presence of a catalytic mixture of (Ph_3_P)AuCl (2 mol %), AgBF_4_ (8 mol %), and H_2_SO_4_ (0.5 mol %) in dioxane (1.0 mL) and water (10 mmol), at 60 °C for 24 h in air. This proceeded efficiently to form a 44:56 mixture of α-diketone **2a** and aldehyde **3a** in 70% combined yield (Scheme 1, Table 1, entry 1). The use of either the gold or silver pre-catalyst alone gave lower yields (Table 1, entries 18 and 19). These results indicate that both the Au source and AgBF_4_ play a crucial role in this oxidation. The superior efficiency of the tetrafluoroborate anion was demonstrated by a comparison with other weakly or non-coordinating counter anions. In addition, a change of the counter anion to OTf^−^, SbF_6_^−^, or NTf_2_^−^ was also effective (Table 1, entries 2–4). The use of other gold catalysts, e.g., (Ph_3_P)AuNO_3_ and IMesAuCl, led to only to combined yields of **2a** and **3a** of 49% and 60%, respectively (Table 1, entries 16–17). Decreasing the amount of the sulfuric acid also resulted in a lower yield, although the addition of a large amount of the acid did not affect the reaction (Table 1, entries 8–9). Different acids were screened (Table 1, entries 1, 5–7) and sulfuric acid was found to be the most effective. The use of solvents such as THF, toluene, DCE or ether resulted in a lower conversion (Table 1, entries 10–13). Treatment of **1a** in an atmosphere of O_2_ (1 atm) afforded **2a** and **3a** in a combined yield of 47% (Table 1, entry 20). When the reaction was conducted under a nitrogen atmosphere, only trace of products were observed (Table 1, entry 21).

**Table 1:** Oxidation of **1a** catalyzed by a mixture of (PPh_3_)AuCl, AgBF_4_, and H_2_SO_4_.^a^

Entry	Au (2 mol %)	Ag (8 mol %)	H_2_O (equiv)	Acid (mol %)	Solvent	Ratio^b^ **2a**:**3a**	Yield (%)^c^ of **2a** and **3a**

1	(PPh_3_)AuCl	AgBF_4_	20	H_2_SO_4_ (0.5)	dioxane	44:56	70
2	(PPh_3_)AuCl	AgOTf	20	H_2_SO_4_ (0.5)	dioxane	38:62	63
3	(PPh_3_)AuCl	AgNTf_2_	20	H_2_SO_4_ (0.5)	dioxane	55:45	43
4	(PPh_3_)AuCl	AgSbF_6_	20	H_2_SO_4_ (0.5)	dioxane	49:51	47
5	(PPh_3_)AuCl	AgBF_4_	20	F_3_CCO_2_H (0.5)	dioxane	43:57	58
6	(PPh_3_)AuCl	AgBF_4_	20	MsOH (0.5)	dioxane	39:61	68
7	(PPh_3_)AuCl	AgBF_4_	20	TsOH (0.5)	dioxane	37:63	48
8	(PPh_3_)AuCl	AgBF_4_	20	H_2_SO_4_ (0.25)	dioxane	49:51	40
9	(PPh_3_)AuCl	AgBF_4_	20	H_2_SO_4_ (1.0)	dioxane	48:52	70
10	(PPh_3_)AuCl	AgBF_4_	20	H_2_SO_4_ (0.5)	THF	47:53	19
11	(PPh_3_)AuCl	AgBF_4_	20	H_2_SO_4_ (0.5)	toluene	39:61	49
12	(PPh_3_)AuCl	AgBF_4_	20	H_2_SO_4_ (0.5)	DCE	43:57	60
13	(PPh_3_)AuCl	AgBF_4_	20	H_2_SO_4_ (0.5)	ether	36:64	37
14	(PPh_3_)AuCl	AgBF_4_	10	H_2_SO_4_ (0.5)	dioxane	46:54	32
15	(PPh_3_)AuCl	AgBF_4_	40	H_2_SO_4_ (0.5)	dioxane	53:47	39
16	(PPh_3_)AuNO_3_	–	20	H_2_SO_4_ (0.5)	dioxane	49:51	49
17	IMeSAuCl	AgBF_4_	20	H_2_SO_4_ (0.5)	dioxane	54:46	60
18	PPh_3_AuCl	–	20	H_2_SO_4_ (0.5)	dioxane	38:62	15
19	–	AgBF_4_	20	H_2_SO_4_ (0.5)	dioxane	47:53	28
20^d^	(PPh_3_)AuCl	AgBF_4_	20	H_2_SO_4_ (0.5)	dioxane	50:50	47
21^e^	(PPh_3_)AuCl	AgBF_4_	20	H_2_SO_4_ (0.5)	dioxane	–	trace

^a^All reactions were carried out using **1a** (0.5 mmol), (Ph_3_P)AuCl (2 mol %), AgBF_4_ (8 mol %), and acid (0.25–1.0 mol %) in solvent (1.0 mL) and water (0.5–1.0 mmol) at 60 °C for 24 h. ^b^The ratio of **2a** and **3a** was determined by GC. ^c^Isolated and combined yield of **2a** and **3a**. ^d^ Under an atmosphere of O_2_ (1 atm). ^e^Under an atmosphere of N_2_.

In order to assess the scope of this process, we examined the oxidation of several aryallenes under the optimized conditions indicated in entry 1 of Table 1. The results are summarized in Table 2. Phenylallene gave a good isolated yield of 1-phenylpropan-1,2-dione (**2c**) and benzaldehyde (**3c**) in a ratio of 43:57 (Table 2, entry 3). With a more electron-donating alkoxy group, the expected products were again obtained in good yields (Table 2, entry 4). In addition, oxidation of arylallene with an electron-withdrawing fluoro or bromo substituent on the benzene ring also took place smoothly (Table 2, entries 5 and 6). Disubstituted allenes were also examined. Thus, the 1,3-disubstituted allene **1g**, was oxidized to afford α-diketone **2g** and aldehyde **3c** in 35% and 32% yields, respectively (Table 2, entry 7). Similarly, oxidation cleavage of 1,1-disubstituted, trisubstituted and tetrasubstituted allenes gave the expected products (Table 2, entries 8–10). In striking contrast to aromatic allenes, aliphatic allenes, such as hepta-1,2-diene and 1-(propa-1,2-dienyl)cyclohex-1-ene failed to undergo Au-catalyzed oxidative transformation under the same reaction conditions.

**Table 2:** Oxidation of **1** catalyzed by a mixture of (PPh_3_)AuCl, AgBF_4_, and H_2_SO_4_.

Entry	Allene **1**	Product **2**	Product **3**	Yield (**2** and **3**) (%)^a^

1	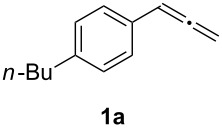	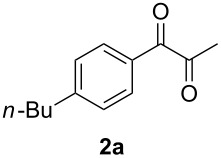	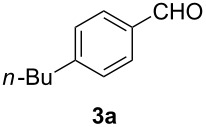	70 (48:52)
2	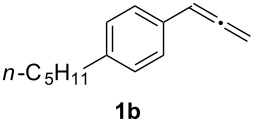	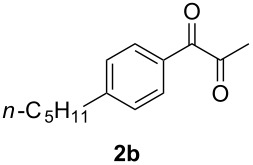	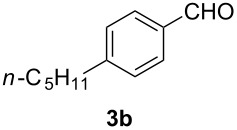	72 (46:57)
3	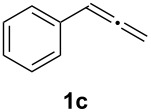	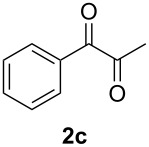	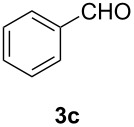	68 (43:57)
4	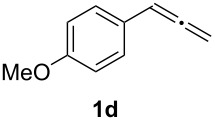	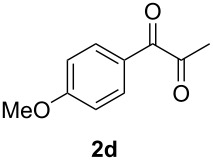	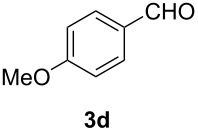	62 (35:65)
5	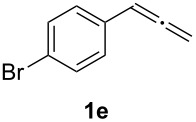	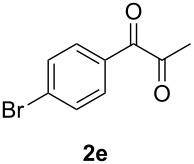	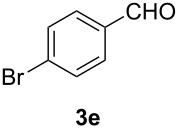	65 (52:48)
6	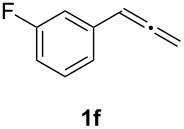	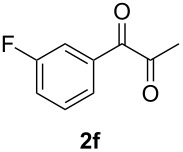	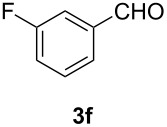	67 (43:57)
7	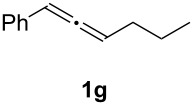	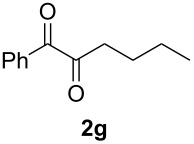	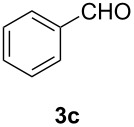	**2g**: 35 **3c**: 32
8	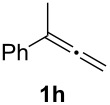	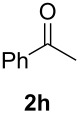		84
9	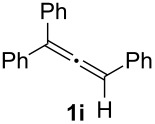	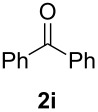	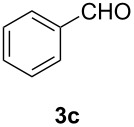	**2i**: 89 **3c**: 85
10	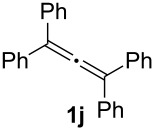	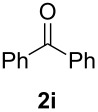		90

^a^Isolated yield. The ratio of **2** and **3** in the parentheses was determined by GC.

**Scheme 2:**
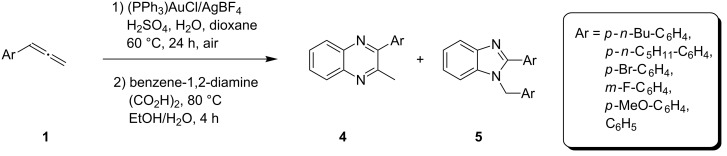
Preparation of quinoxalines and benzimidazoles.

Having prepared a variety of α-diketones and aldehydes successfully, we then undertook the synthesis of quinoxalines and benzimidazoles (Scheme 2). Thus, the treatment of the corresponding mixture of α-diketone **2** and aldehyde **3** with benzene-1,2-diamine in the presence of 20 mol % oxalic acid afforded the desired quinoxalines **4** and benzimidazoles **5** in high yields (Table 3, entries 1–6).

**Table 3:** Preparation of quinoxalines and benzimidazoles.

Entry	Allene **1**	Ratio (**2**: **3**)^a^	Product **4**	Yield (%)^b^	Product **5**	Yield (%)^b^

1	**1a**	48:52	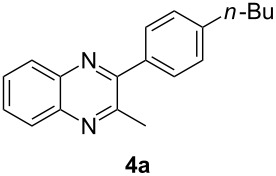	97	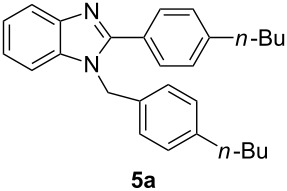	94
2	**1b**	46:57	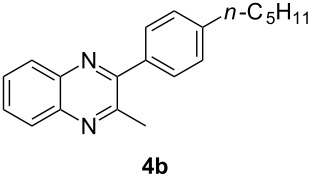	95	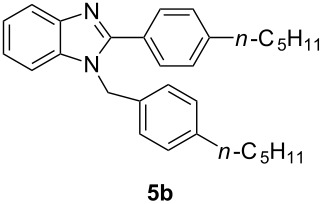	90
3	**1c**	43:57	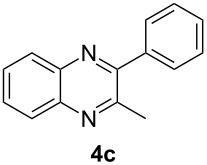	97	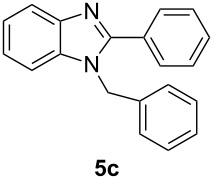	94
4	**1d**	35:65	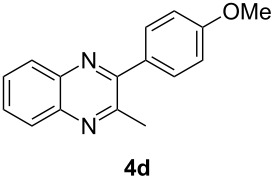	90	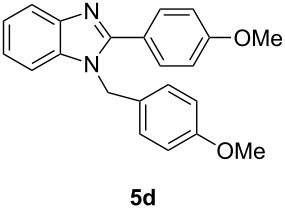	92
5	**1e**	52:48	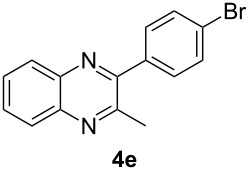	97	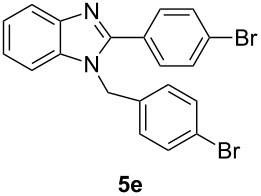	92
6	**1f**	43:57	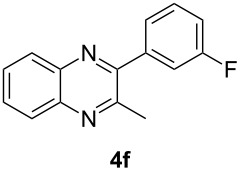	97	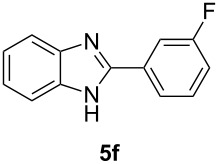	94

^a^The ratio of **2** and **3** was determined by GC. ^b^Isolated yield.

## Conclusion

We have developed a new gold-catalyzed oxidation of arylallenes to give α-diketones and aldehydes in good yields. In addition, the directed synthesis of quinoxalines and benzimidazoles via the condensation of the resulting α-diketones and aldehydes with benzene-1,2-diamine was achieved in high yields. This reaction appears to proceed via oxidation/hydration and oxidative cleavage of the allene, and investigations into the mechanism of this reaction are underway in our laboratory.

## Experimental

General methods: Unless otherwise noted, materials were obtained from commercial suppliers and used without further purification. Thin layer chromatography (TLC) was performed on silica gel 60 F_254_ and visualized with UV light. Column chromatography was performed with silica gel (mesh 300–400). ^1^H NMR and ^13^C NMR spectra were recorded on a Bruker Avance 500 MHz spectrometer in CDCl_3_ with Me_4_Si as internal standard. Data are reported as follows: Chemical shift in ppm (δ), multiplicity (s = singlet, d = doublet, t = triplet, q = quartet, br = broad and m = multiplet), coupling constant in Hertz (Hz) and signal integration. Infrared spectra (IR) were obtained on 370 FT-IR spectrometer; absorptions are reported in cm^−1^. Mass spectra were obtained under electron impact mode (EI) and high resolution mass spectra were measured on a high resolution mass spectrometer (GCT Premier).

### General procedure

Step A (a typical procedure): Sulfuric acid (0.5 mol %) was added to a mixture of 4-butylphenylallene (0.5 mmol), water (10 mmol), (PPh_3_)AuCl (2 mol %), AgBF_4_ (8 mol %), and dioxane (1 mL). The mixture was stirred at 60 °C for 24 , the reaction quenched with a saturated solution of NaHCO_3_ and extracted with ethyl acetate (3 × 10 mL). The combined organic layers were washed with brine, dried over Na_2_SO_4_ and concentrated in vacuo. The residue was purified by flash chromatography to give the desired products **2a** and **3a** (48:52, 63.75 mg, 70%).

Step B (a typical procedure): A mixture of **2a** and **3a** (63.75 mg), benzene-1,2-diamine (28 mg, 0.259 mmol), oxalic acid (6.3 mg, 0.07 mmol, 20 mol %), water (1 mL) was dissolved in ethanol (1 mL). The mixture was heated under reflux for 4 h. The reaction was quenched with a saturated solution of NaHCO_3_ and then extracted with ethyl acetate (3 × 10 mL). The combined organic layers were washed with brine, dried over Na_2_SO_4_ and concentrated in vacuo. The residue was purified by flash chromatography to give the desired products **4a** (45.0 mg, 97%) and **5a** (33.9 mg, 94%).

## Supporting Information

File 1 Analytical and spectroscopic data for new compounds.
