# Supplementary material for: Gold-catalyzed oxidation of arylallenes: Synthesis of quinoxalines and benzimidazoles
Source: Beilstein J Org Chem. 2011 Jun 24;7:860–5. doi: 10.3762/bjoc.7.98 (PMC3135117; doi:10.3762/bjoc.7.98)
Supplement: File 1 — Analytical and spectroscopic data for new compounds. [file Beilstein_J_Org_Chem-07-860-s001.pdf]

Supporting Information

for

Gold-catalyzed oxidation of arylallenes: Synthesis of quinoxalines and benzimidazoles

Dong-Mei Cui*[1], Dan-Wen Zhuang[1], Ying Chen[1] and Chen Zhang[2]

Address: [1] College of Pharmaceutical Science, Zhejiang University of Technology, Hangzhou 310014, PR China and [2] College of Pharmaceutical Sciences, Zhejiang University, Hangzhou 310058, PR China

Email: Dong-Mei Cui* - cuidongmei@zjut.edu.cn

* Corresponding author

Analytical and spectroscopic data for new compounds

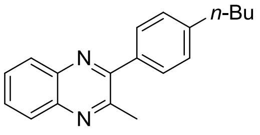

4a

2-(4-butylphenyl)-3-methylquinoxaline (4a): Orange oil; ^{1}H NMR ($CDCl_3$, 500 MHz) δ 8.13-8.05 (m, 2H), 7.76-7.70 (m, 2H), 7.59 (d, *J* = 8.0 Hz, 2H), 7.35 (d, *J* = 8.0 Hz, 2H), 2.81 (s, 3H), 2.72 (t, *J* = 7.5 Hz, 2H), 1.70-1.64 (m, 2H), 1.45-1.37 (m, 2H), 0.97 (t, *J* = 7.5 Hz, 3H); ^{13}C NMR ($CDCl_3$, 100 MHz) δ 155.00, 152.63, 143.99, 141.09, 141.04, 136.31, 129.53, 029.17, 129.11, 128.86, 128.61, 128.24, 35.47, 33.50, 24.42, 22.30, 13.92; IR (cm^{-1}, KBr) 3421, 2955, 1634, 1384, 761. HRMS (EI) calcd for $C_{19}H_{20}N_2$ 276.1626, Found 276.1601.

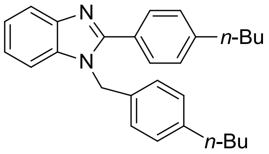

5a

1-(4-butylbenzyl)-2-(4-butylphenyl)-1*H*-benzimidazole (5a): Yellow oil; ^{1}H NMR ($CDCl_3$, 500 MHz) δ 7.86-7.84 (m, 1H), 7.62-7.61 (m, 2H), 7.30-7.20 (m, 5H), 7.13-7.12 (m, 2H), 7.02-7.00 (m, 2H), 5.42 (s, 2H), 2.67-2.57 (m, 4H), 1.63-1.56 (m, 4H), 1.38-1.24(m, 4H), 0.94-0.91 (m, 6H); ^{13}C NMR ($CDCl_3$, 125 MHz) δ 154.39, 145.01, 143.13, 142.48, 136.10, 133.64, 129.17, 129.03, 128.82, 127.31, 125.91, 122.80, 122.53, 119.80, 110.56, 48.25, 35.50, 35.26, 33.56, 33.39, 22.38, 22.30, 13.95 (2C); IR (cm^{-1}, KBr) 3461, 2957, 1741, 1615, 1384, 1243, 745. HRMS (EI) calcd for $C_{28}H_{32}N_2$ 396.2565, Found 396.2569

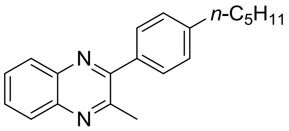

4b

2-methyl-3-(4-pentylphenyl)quinoxaline (4b): Yellow oil; ^{1}H NMR ($CDCl_3$, 500 MHz) δ 8.13-8.11 (m, 2H), 8.07-8.05 (m, 2H), 7.76-7.70 (m, 2H), 7.59 (d, *J* = 8.0 Hz, 2H), 7.35 (d, *J* = 8.0 Hz, 2H), 2.81 (s, 3H), 2.70 (t, *J* = 7.5 Hz, 2H), 1.71-1.65 (m, 2H), 1.38-1.36 (m, 2H), 0.92 (t, *J* = 7.0 Hz, 3H); ^{13}C NMR ($CDCl_3$, 125 MHz): δ 155.0, 152.6, 144.0, 141.1, 141.0, 136.3, 129.5, 129.2, 129.1, 128.9, 128.6,

128.3, 35.8, 31.5, 31.1, 24.5, 22.6, 14.0; IR (cm^{-1}, KBr) 3455, 2929, 2856, 1614, 1341, 910, 735. HRMS (EI) calcd for $C_{20}H_{22}N_2$ 290.1783, Found 290.1779

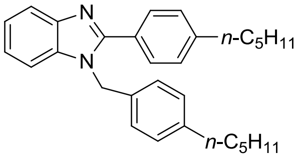

5b

1-(4-pentylbenzyl)-2-(4-pentylphenyl)-1*H*-benzimidazole (5b): Yellow oil; ^{1}H NMR ($CDCl_3$, 500 MHz) δ 7.87 (d, *J* = 8.0 Hz, 1H), 7.63 (d, *J* = 8.0 Hz, 2H), 7.31-7.22 (m, 5H), 7.15 (d, *J* = 8.0 Hz, 2H), 7.03 (d, *J* = 8.0 Hz, 2H), 5.45 (s, 2H), 2.67 (t, *J* = 8.0 Hz, 2H), 2.60 (t, *J* = 8.0 Hz, 2H), 1.67-1.61 (m, 4H), 1.35-1.21 (m, 8H), 0.91 (t, *J* = 7.0 Hz, 6H); ^{13}C NMR ($CDCl_3$, 125 MHz): δ 154.4, 145.0, 143.2, 142.5, 136.1, 133.7, 129.2, 129.0, 128.8, 127.4, 125.9, 122.8, 122.5, 119.8, 110.5, 48.3, 35.8, 35.5, 31.5, 31.4, 31.1, 30.9, 30.3, 29.7, 22.5, 14.0; IR (cm^{-1}, KBr) 3416, 2927, 1616, 1384, 912, 743. HRMS (EI) calcd for $C_{30}H_{36}N_2$ 424.2878, Found 424.2875

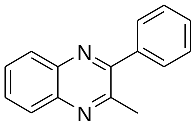

4c

2-methyl-3-phenylquinoxaline (4c) [1]: Yellow oil; ^{1}H NMR ($CDCl_3$, 500 MHz): δ 8.13-8.10 (m, 1H), 8.07-8.05 (m, 1H), 7.75-7.71 (m, 2H), 7.67-7.64 (m, 2H), 7.55-7.49 (m, 3H), 2.81 (s, 3H).

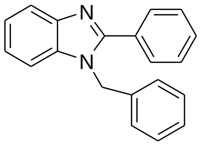

5c

1-benzyl-2-phenyl-1*H*-benzimidazole (5c) [2]: White solid; ^{1}H NMR ($CDCl_3$,500 MHz): δ 7.87 (d, *J* = 8.0, 1H), 7.70-7.68 (m, 2H), 7.48-7.43 (m, 3H), 7.35-7.28 (m, 4H), 7.26-7.22 (m, 2H), 7.21-7.10 (m, 2H), 5.46 (s, 2H).

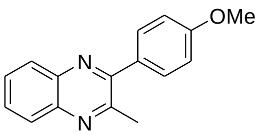

4d

2-(4-methoxyphenyl)-3-methylquinoxaline (4d) [3]: Yellow oil; ^{1}H NMR (CDCl$_3$, 500 MHz): δ 8.11-8.07 (m, 1H), 8.05-8.02 (m, 1H), 7.72-7.71 (m, 2H), 7.65-7.62 (m, 2H), 7.07-7.03 (m, 2H), 3.89 (s, 3H), 2.80 (s, 3H).

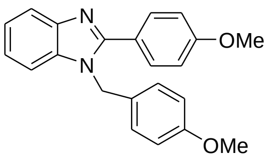

5d

1-(4-methoxybenzyl)-2-(4-methoxyphenyl)-1*H*-benzimidazole (5d) [2]: Yellow solid; ^{1}H NMR (CDCl$_3$, 500 MHz):δ 7.87 (d, J = 8.0 Hz, 1H), 7.70-7.68 (m, 2H), 7.48-7.43 (m, 3H), 7.35-7.28 (m, 2H), 7.26-7.22 (m, 2H), 7.21-7.10 (m, 2H), 5.46 (s, 2H), 3.89 (s, 3H), 3.66 (s, 3H).

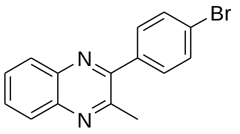

4e

2-(4-bromophenyl)-3-methylquinoxaline (4e) [4]: Orange oil; ^{1}H NMR (CDCl$_3$, 500 MHz): δ 8.12-8.06 (m, 2H), 7.79-7.72 (m, 2H), 7.70-7.67 (m, 2H), 7.58-7.55 (m, 2H), 2.79 (s, 3H).

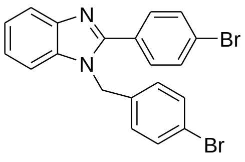

5e

1-(4-bromobenzyl)-2-(4-bromophenyl)-1*H*-benzo[d]imidazole (5e) [2]: Yellow solid; mp 160°C; ^{1}H NMR (CDCl$_3$, 500 MHz): δ 7.87 (d, J = 8.0 Hz, 1H), 7.70-7.68 (m, 2H), 7.48-7.43 (m, 3H), 7.35-7.28 (m, 2H), 7.26-7.22 (m, 2H), 7.21-7.10 (m, 2H), 5.46 (s, 2H).

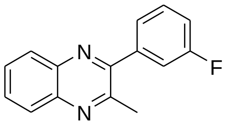

4f

2-(3-fluorophenyl)-3-methylquinoxaline (4f): White solid, mp 85-87℃; ^{1}H NMR (CDCl$_3$, 500 MHz) δ 8.13-8.06 (m, 2H), 7.79-7.73 (m, 2H), 7.54-7.38 (m, 3H), 7.23-7.19 (m, 1H), 2.80 (s, 3H); ^{13}C NMR

(CDCl$_3$, 125 MHz): δ 162.79 (d, *J* = 246.25 Hz), 153.53, 153.51, 152.23, 141.45, 141.17 (d, *J* = 6.3 Hz), 140.92, 130.27 (d, *J* = 8.8 Hz), 130.13, 129.40 (d, *J* = 26.3 Hz), 128.42, 124.78 (d, *J* = 2.5 Hz), 116.35, 116.09 (d, *J* = 20.0 Hz), 24.31; IR (cm^{-1}, KBr) 3417, 2982, 1740, 1616, 1240, 1048, 762. HRMS (EI) calcd for $C_{15}H_{11}FN_2$ 238.0906, Found 238.0894.

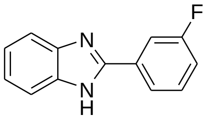

5f

2-(3-fluorophenyl)-1*H*-benzimidazole (5f): White solid, mp 115-116℃; ^{1}H NMR (CDCl$_3$, 500 MHz): δ 7.83 (d, *J* = 8.0 Hz, 1H), 7.81-7.78 (m, 1H), 7.67-7.65 (m, 2H), 7.46-7.42 (m, 1H), 7.32-7.29 (m, 2H), 7.17-7.14 (m, 1H); ^{13}C NMR (DMSO, 125 MHz): δ 162.92 (d, *J* = 241.3 Hz), 150.39, 144.08, 135.41, 132.93 (d, *J* = 7.5 Hz), 131.64 (d, *J* = 8.8 Hz), 123.41, 122.97 (d, *J* = 2.5 Hz), 122.39, 119.54, 117.08 (d, *J* = 20.0 Hz), 113.46 (d, *J* = 22.5 Hz), 111.96; IR (cm^{-1}, KBr) 3422, 2994, 1770, 1618, 1383, 1246, 1050. HRMS (EI) calcd for $C_{13}H_9FN_2$ 212.0750, Found 212.0764.

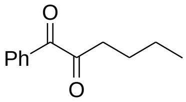

2g

1-phenylhexane-1,2-dione (2g) [5]: Yellow oil; ^{1}H NMR(CDCl$_3$, 500 MHz):δ 7.57-7.53 (m, 2H), 7.40-7.25 (m,3H), 2.64 (t, *J* = 7.5 Hz, 3H), 1.73- 1.68 (m, 2H), 0.97 (t, *J* = 7.5 Hz, 3H).

References

1. More, S. V.; Sastry, M. N. V.; Yao, C.-F. *Green Chem*. **2006**, *8*, 91. doi:10.1039/b510677j

2. Wan, J.-P.; Gan, S.-F.; Wu, J.-M.; Pan, Y.-J. *Green Chem*., **2009**, *11*,1633. doi:10.1039/b914286j

3. Hahn, W. E.; Lesiak, J. *Pol. J. Chem*. **1985**, *59*, 627.

4. Lee, D. G.; Chang, V. S. *Synthesis* **1978,** 462. doi:10.1055/s-1978-24783

5. Ren, W.; Liu, J.-F.; Chen, L.; Wan, X.-B. *Adv. Synth. Catal*. **2010**, *352*, 1424. doi:10.1002/adsc.201000250

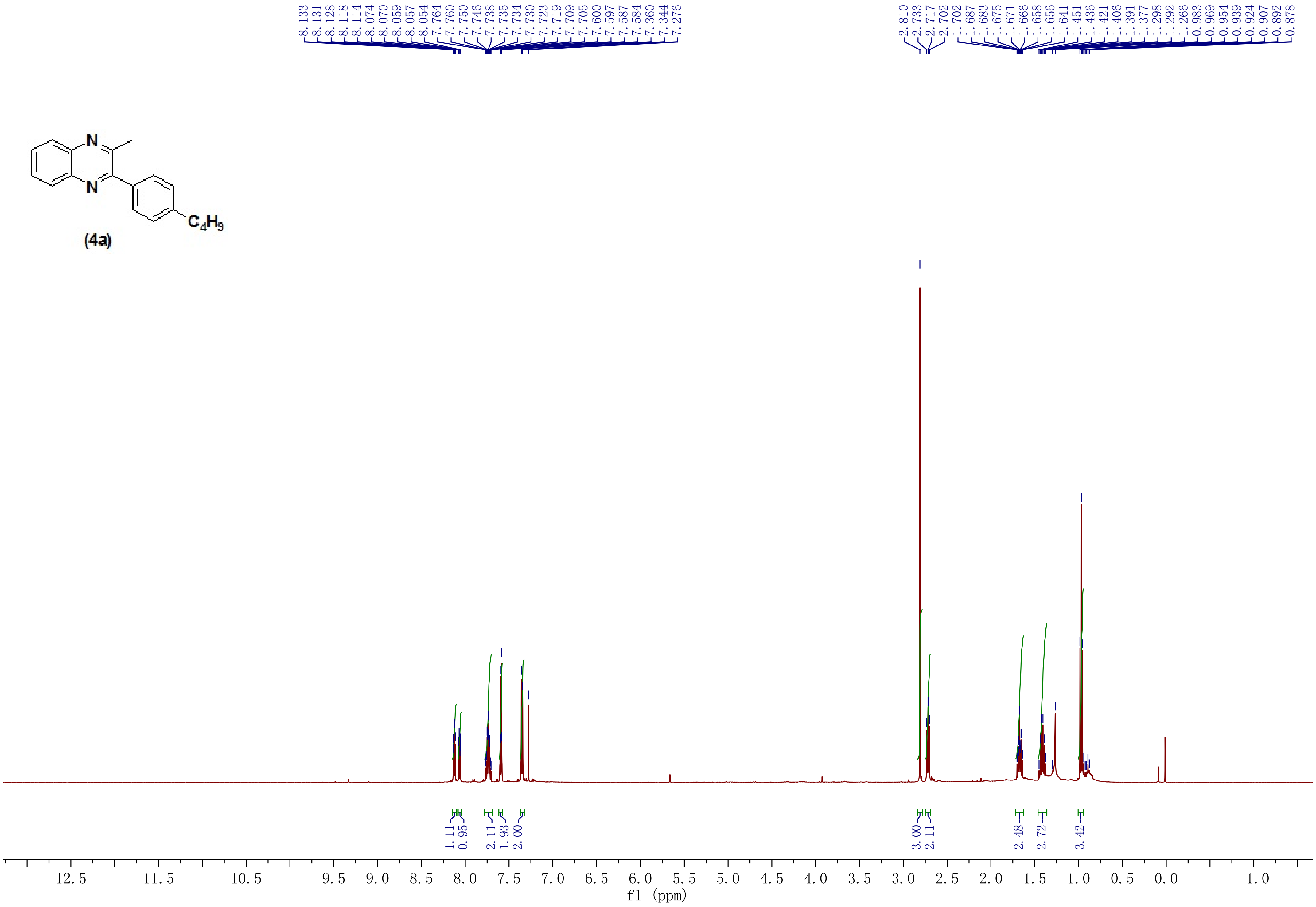
8.133
8.131
8.128
8.118
8.114
8.074
8.070
8.059
8.057
8.054
7.764
7.760
7.750
7.746
7.738
7.735
7.734
7.730
7.723
7.719
7.709
7.705
7.600
7.597
7.587
7.584
7.360
7.344
7.276
2.810
2.733
2.717
2.702
1.702
1.687
1.683
1.675
1.671
1.666
1.658
1.656
1.641
1.451
1.436
1.421
1.406
1.391
1.377
1.298
1.292
1.266
0.983
0.969
0.954
0.939
0.924
0.907
0.892
0.878
N
N
C_4H_9
(4a)
1.11
0.95
2.11
1.93
2.00
3.00
2.11
2.48
2.72
3.42
12.5
11.5
10.5
9.5
9.0
8.5
8.0
7.5
7.0
6.5
6.0
5.5
5.0
4.5
4.0
3.5
3.0
2.5
2.0
1.5
1.0
0.5
0.0
-1.0
f1 (ppm)

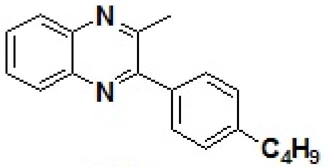

(4a)

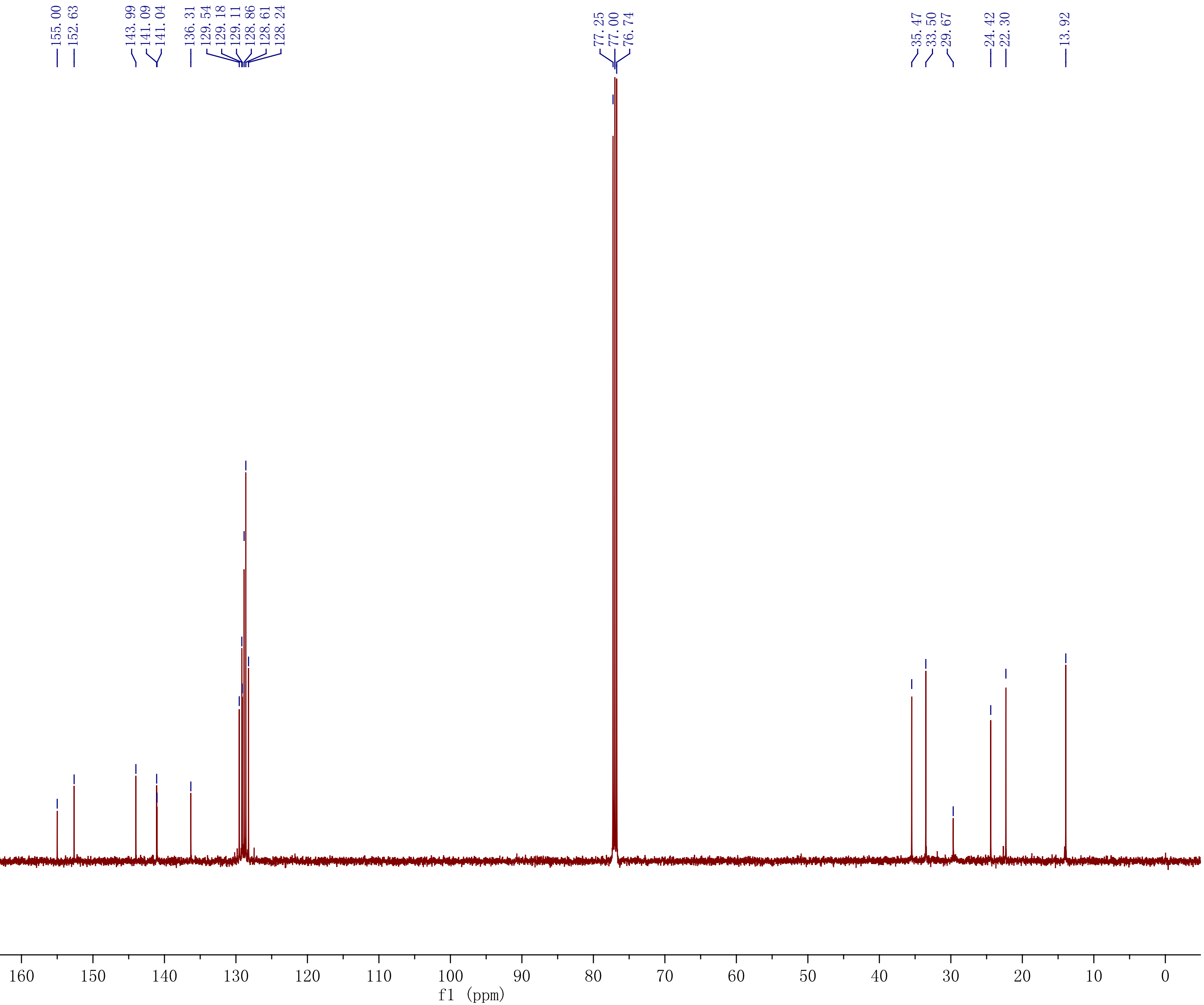
155.00
152.63
143.99
141.09
141.04
136.31
129.54
129.18
129.11
128.86
128.61
128.24
77.25
77.00
76.74
35.47
33.50
29.67
24.42
22.30
13.92
190
180
170
160
150
140
130
120
110
100
90
80
70
60
50
40
30
20
10
0
f1 (ppm)

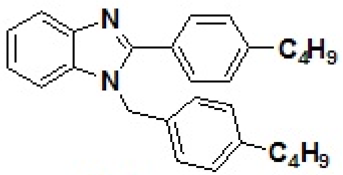
N
N
C4H9
C4H9

(5a)

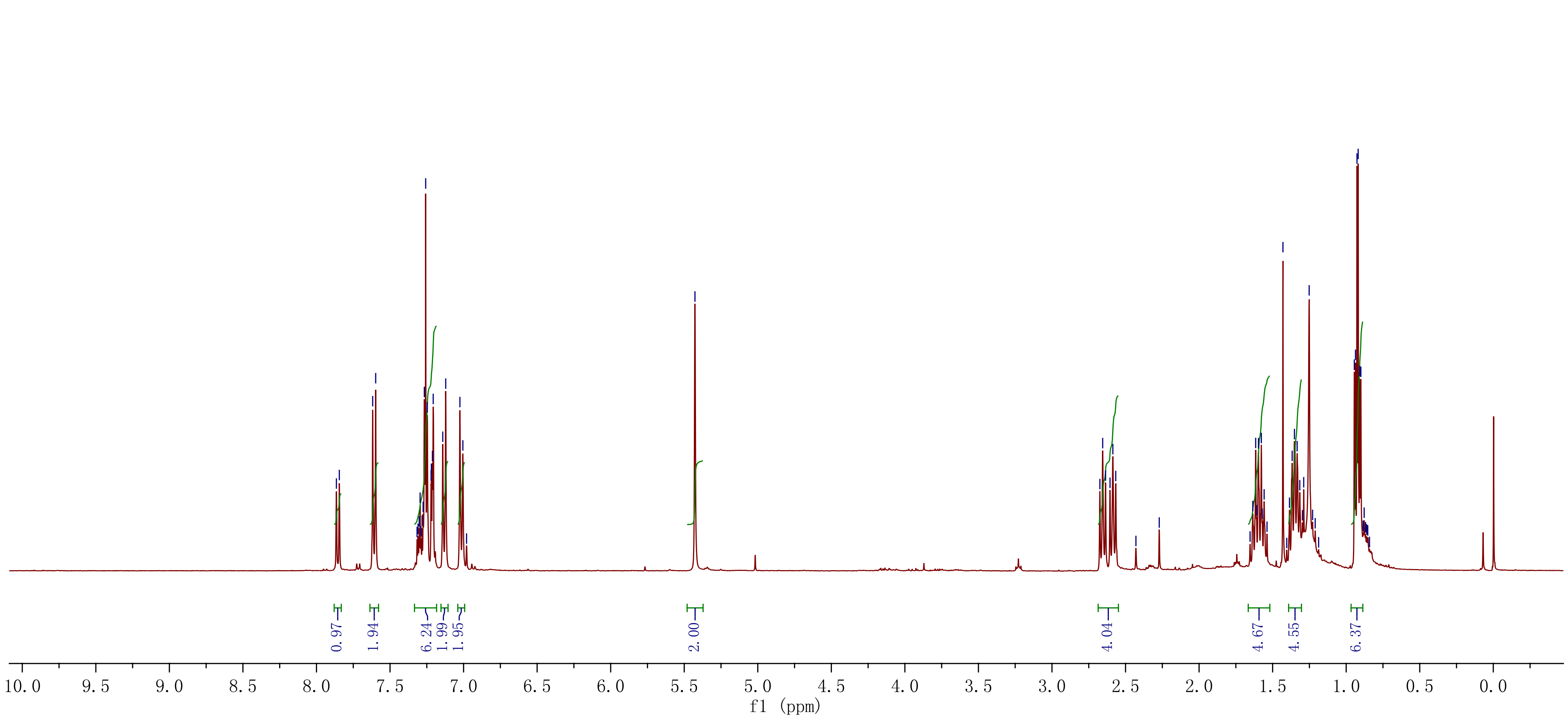
7.865
7.845
7.618
7.598
7.316
7.310
7.302
7.296
7.289
7.282
7.275
7.267
7.258
7.247
7.221
7.219
7.212
7.206
7.142
7.121
7.025
5.427
2.674
2.655
2.636
2.605
2.586
2.567
2.429
2.271
1.615
1.596
1.577
1.430
1.367
1.352
1.333
1.289
1.252
0.937
0.927
0.919
0.908
0.900
0.885
0.878
0.868
0.860
0.853
0.842
0.97
1.94
6.24
1.99
1.95
2.00
4.04
4.67
4.55
6.37
10.0
9.5
9.0
8.5
8.0
7.5
7.0
6.5
6.0
5.5
5.0
4.5
4.0
3.5
3.0
2.5
2.0
1.5
1.0
0.5
0.0
f1 (ppm)

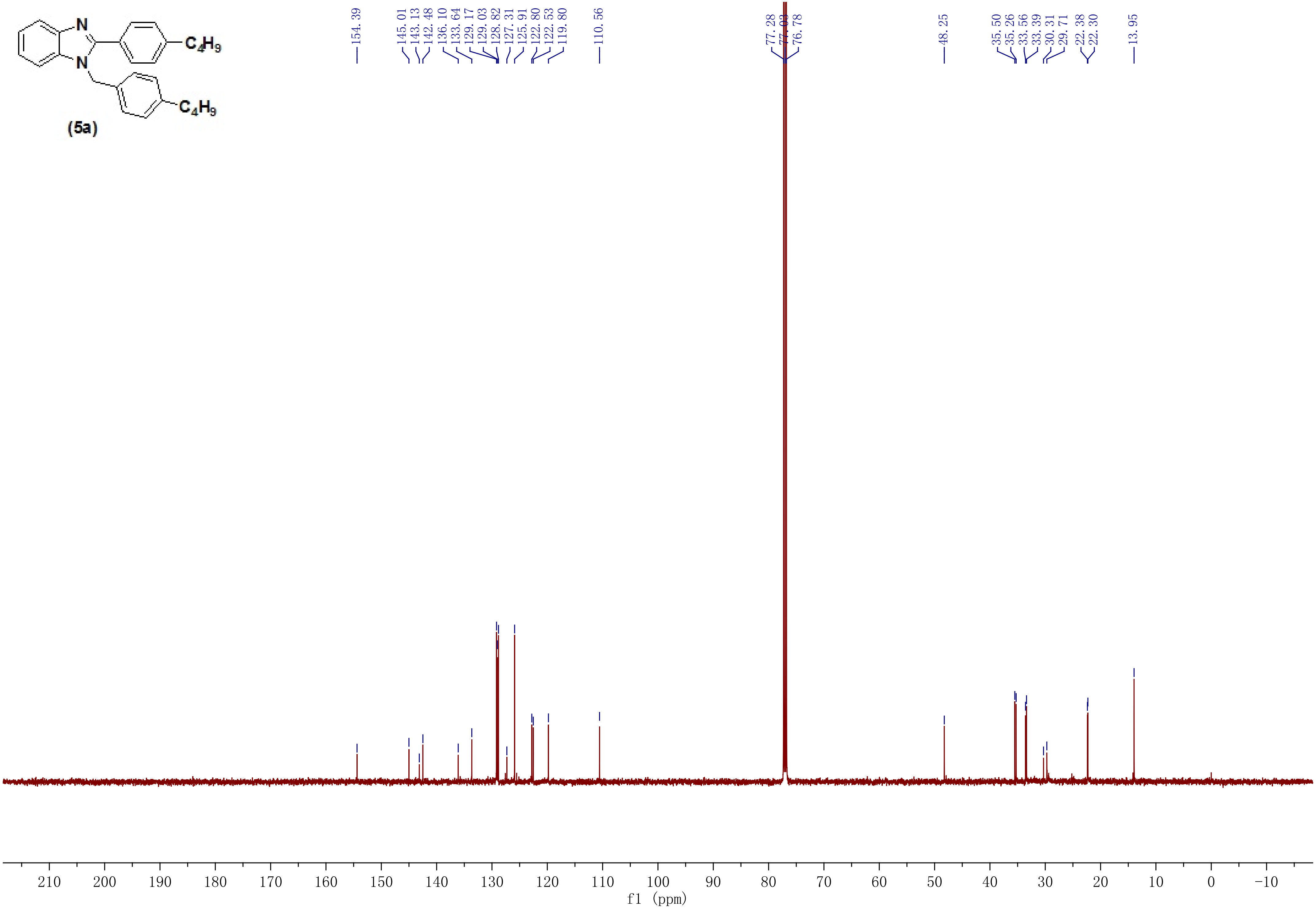

N
N
C_4H_9
C_4H_9
(5a)
154.39
145.01
143.13
142.48
136.10
133.64
129.17
129.03
128.82
127.31
125.91
122.80
122.53
119.80
110.56
77.28
77.03
76.78
48.25
35.50
35.26
33.56
33.39
30.31
29.71
22.38
22.30
13.95
210 200 190 180 170 160 150 140 130 120 110 100 90 80 70 60 50 40 30 20 10 0 −10
f1 (ppm)

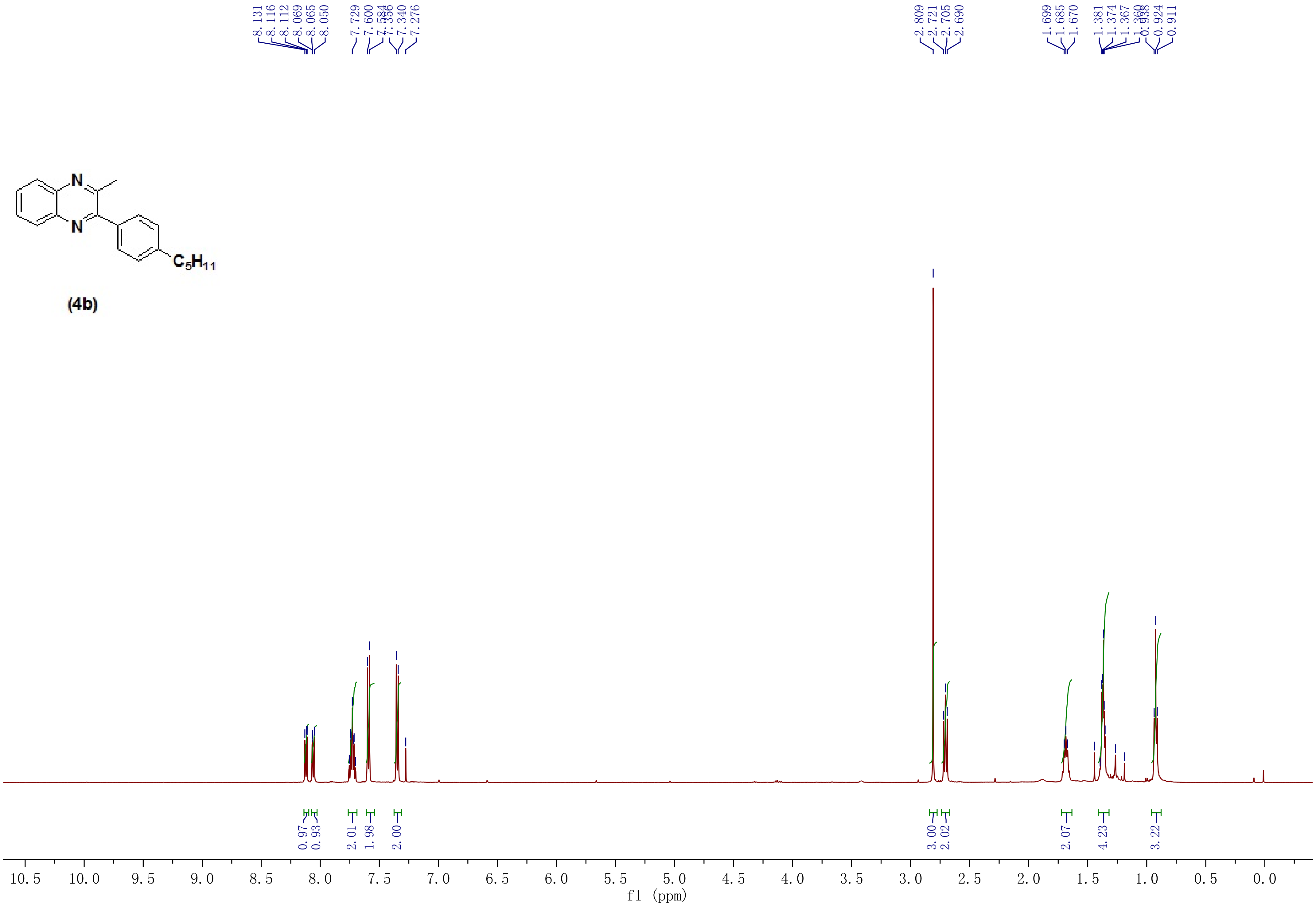

8.131
8.116
8.112
8.069
8.065
8.050
7.729
7.600
7.356
7.340
7.276
2.809
2.721
2.705
2.690
1.699
1.685
1.670
1.381
1.374
1.367
0.924
0.911
N
N
C5H11
(4b)
0.97
0.93
2.01
1.98
2.00
3.00
2.02
2.07
4.23
3.22
10.5
10.0
9.5
9.0
8.5
8.0
7.5
7.0
6.5
6.0
5.5
5.0
4.5
4.0
3.5
3.0
2.5
2.0
1.5
1.0
0.5
0.0
f1 (ppm)

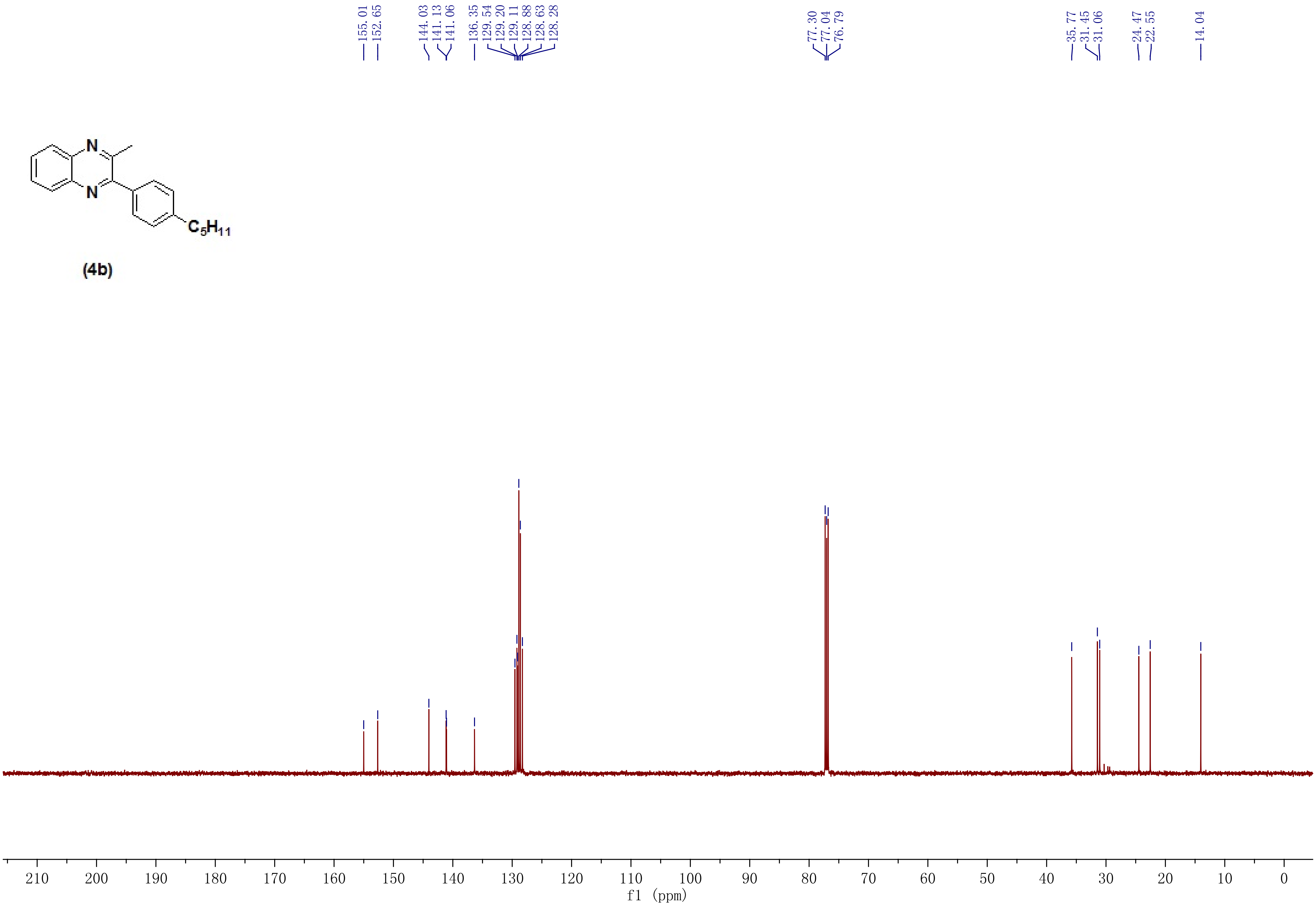

N
N
C5H11
155.01
152.65
144.03
141.13
141.06
136.35
129.54
129.20
129.11
128.88
128.63
128.28
77.30
77.04
76.79
35.77
31.45
31.06
24.47
22.55
14.04
210
200
190
180
170
160
150
140
130
120
110
100
90
80
70
60
50
40
30
20
10
0
f1 (ppm)

(4b)

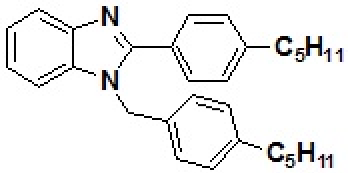
N
N
C5H11
C5H11

(5b)

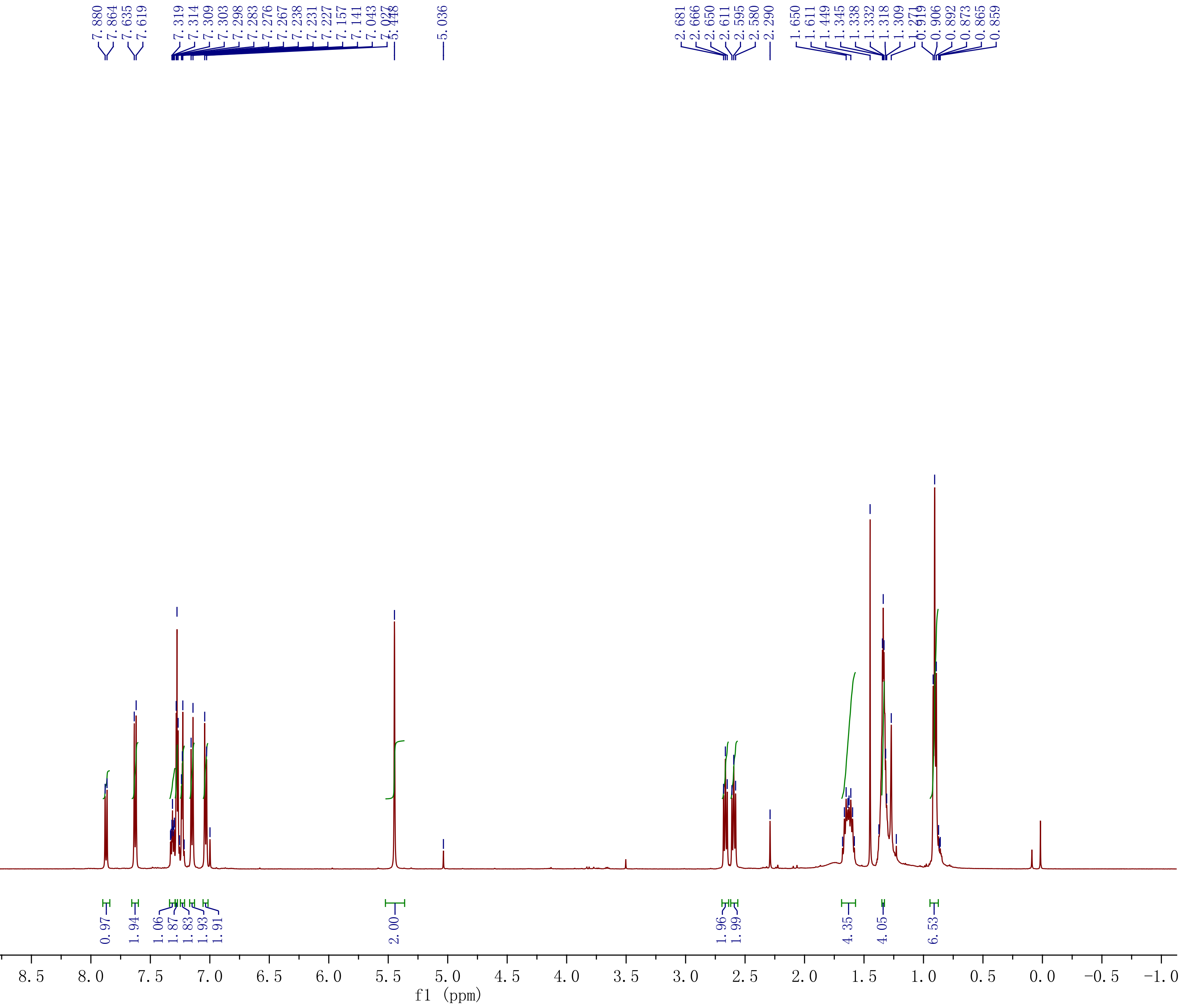
7.880
7.864
7.635
7.619
7.319
7.314
7.309
7.303
7.298
7.283
7.276
7.267
7.238
7.231
7.227
7.157
7.141
7.043
5.036
2.681
2.666
2.650
2.611
2.595
2.580
2.290
1.650
1.611
1.449
1.345
1.338
1.332
1.318
1.309
1.271
0.906
0.892
0.873
0.865
0.859
0.97
1.94
1.06
1.87
1.83
1.93
1.91
2.00
1.96
1.99
4.35
4.05
6.53
11.0 10.5 10.0 9.5 9.0 8.5 8.0 7.5 7.0 6.5 6.0 5.5 5.0 4.5 4.0 3.5 3.0 2.5 2.0 1.5 1.0 0.5 0.0 -0.5 -1.0
f1 (ppm)

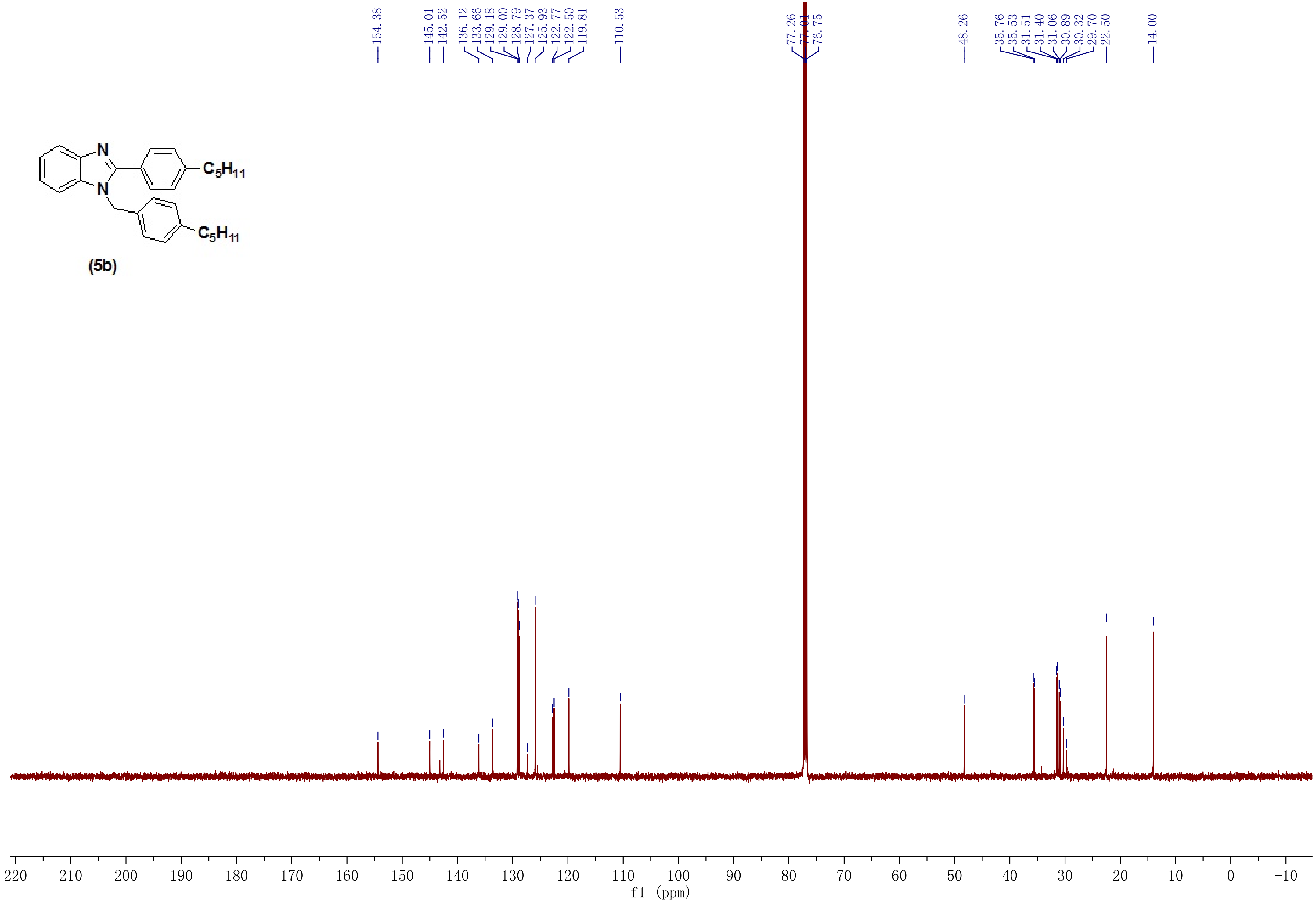

N
N
C5H11
C5H11
(5b)
154.38
145.01
142.52
136.12
133.66
129.18
129.00
128.79
127.37
125.93
122.77
122.50
119.81
110.53
77.26
77.01
76.75
48.26
35.76
35.53
31.51
31.40
31.06
30.89
30.32
29.70
22.50
14.00
220 210 200 190 180 170 160 150 140 130 120 110 100 90 80 70 60 50 40 30 20 10 0 −10
f1 (ppm)

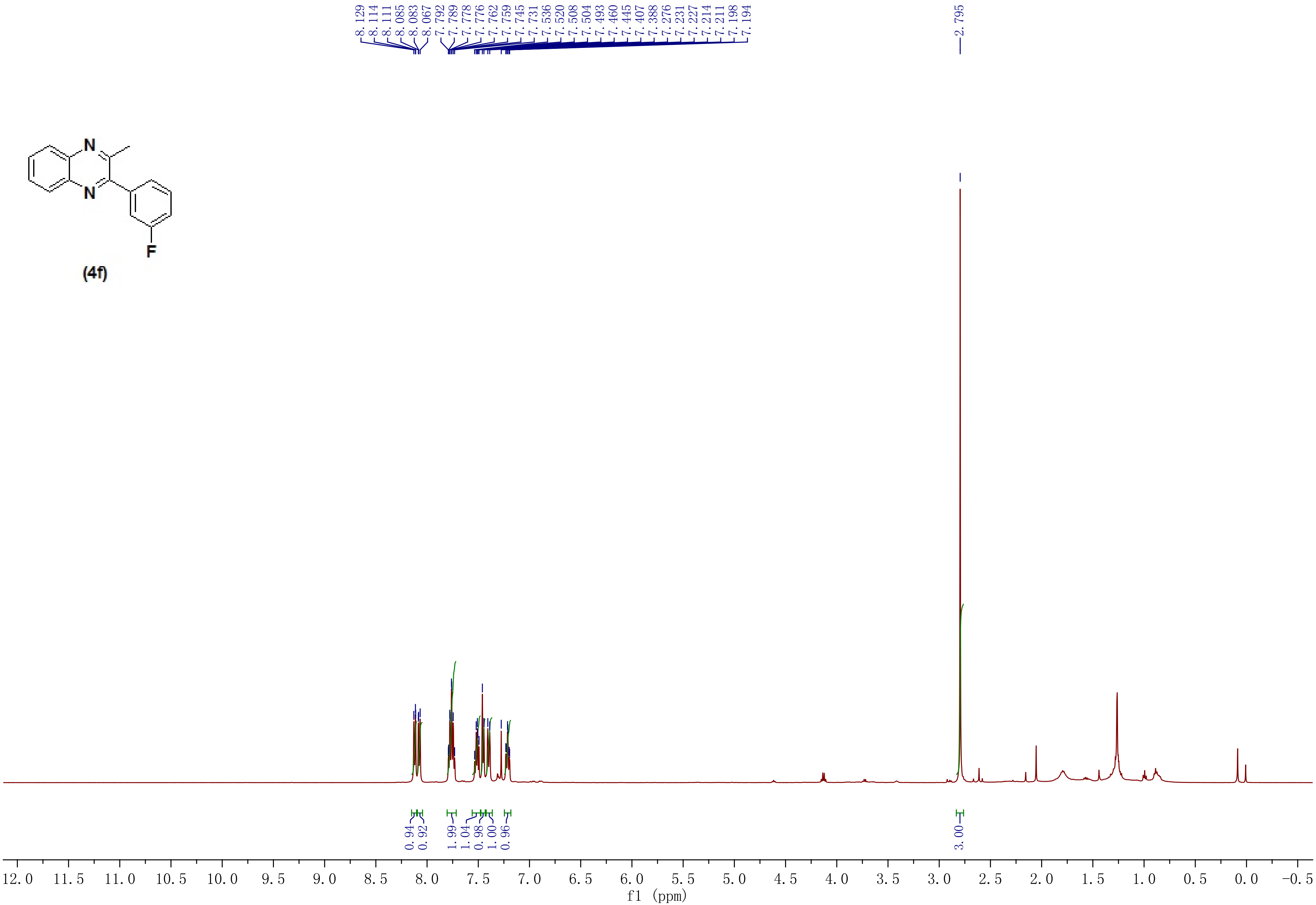
N
N
F
(4f)
8.129
8.114
8.111
8.085
8.083
8.067
7.792
7.789
7.778
7.776
7.762
7.759
7.745
7.731
7.536
7.520
7.508
7.504
7.493
7.460
7.445
7.407
7.388
7.276
7.231
7.227
7.214
7.211
7.198
7.194
2.795
0.94
0.92
1.99
1.04
0.98
1.00
0.96
3.00
12.0 11.5 11.0 10.5 10.0 9.5 9.0 8.5 8.0 7.5 7.0 6.5 6.0 5.5 5.0 4.5 4.0 3.5 3.0 2.5 2.0 1.5 1.0 0.5 0.0 -0.5
f1 (ppm)

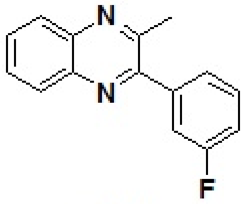
N
N
F

(4f)

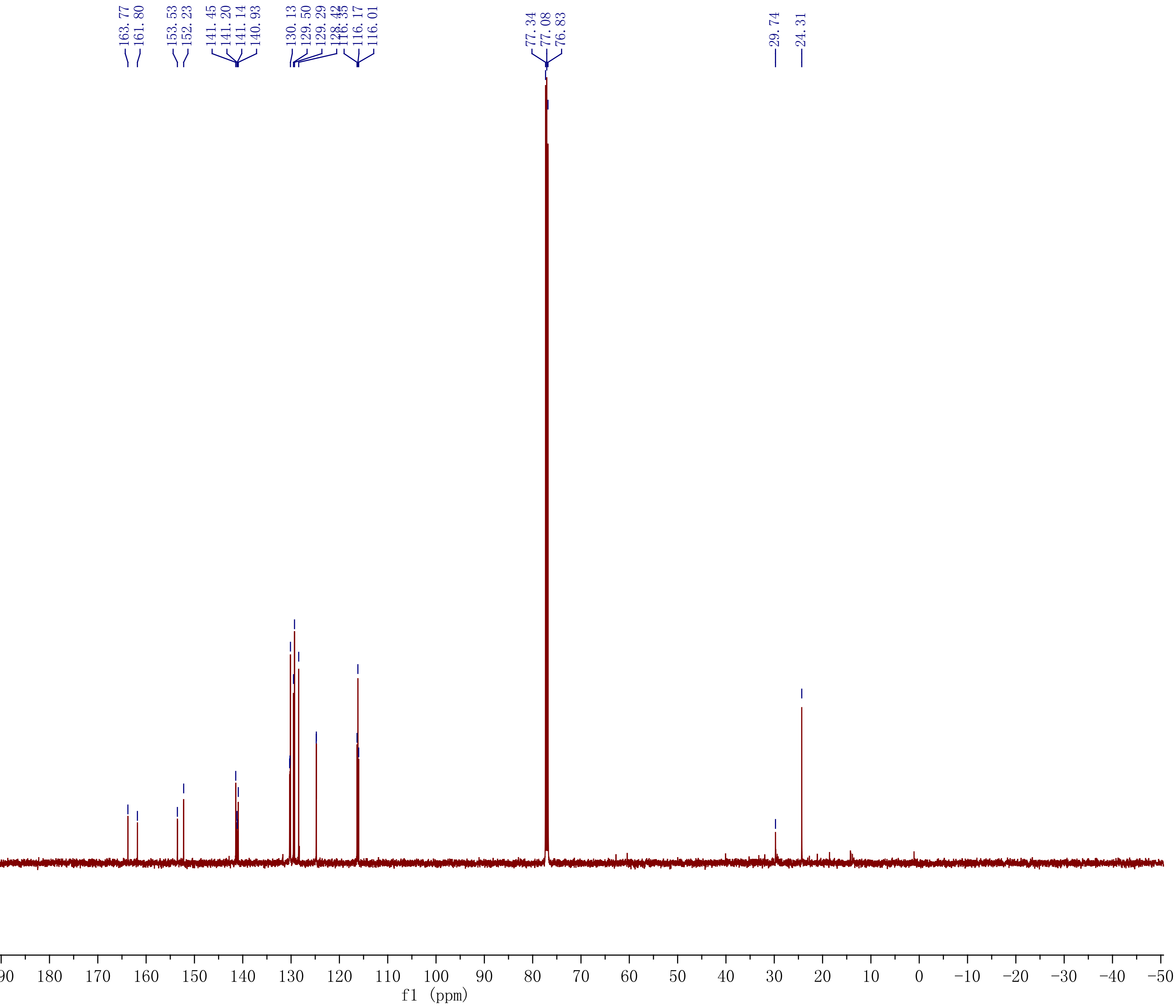
163.77
161.80
153.53
152.23
141.45
141.20
141.14
140.93
130.13
129.50
129.29
116.35
116.17
116.01
77.34
77.08
76.83
29.74
24.31
250 240 230 220 210 200 190 180 170 160 150 140 130 120 110 100 90 80 70 60 50 40 30 20 10 0 −10 −20 −30 −40 −50
f1 (ppm)

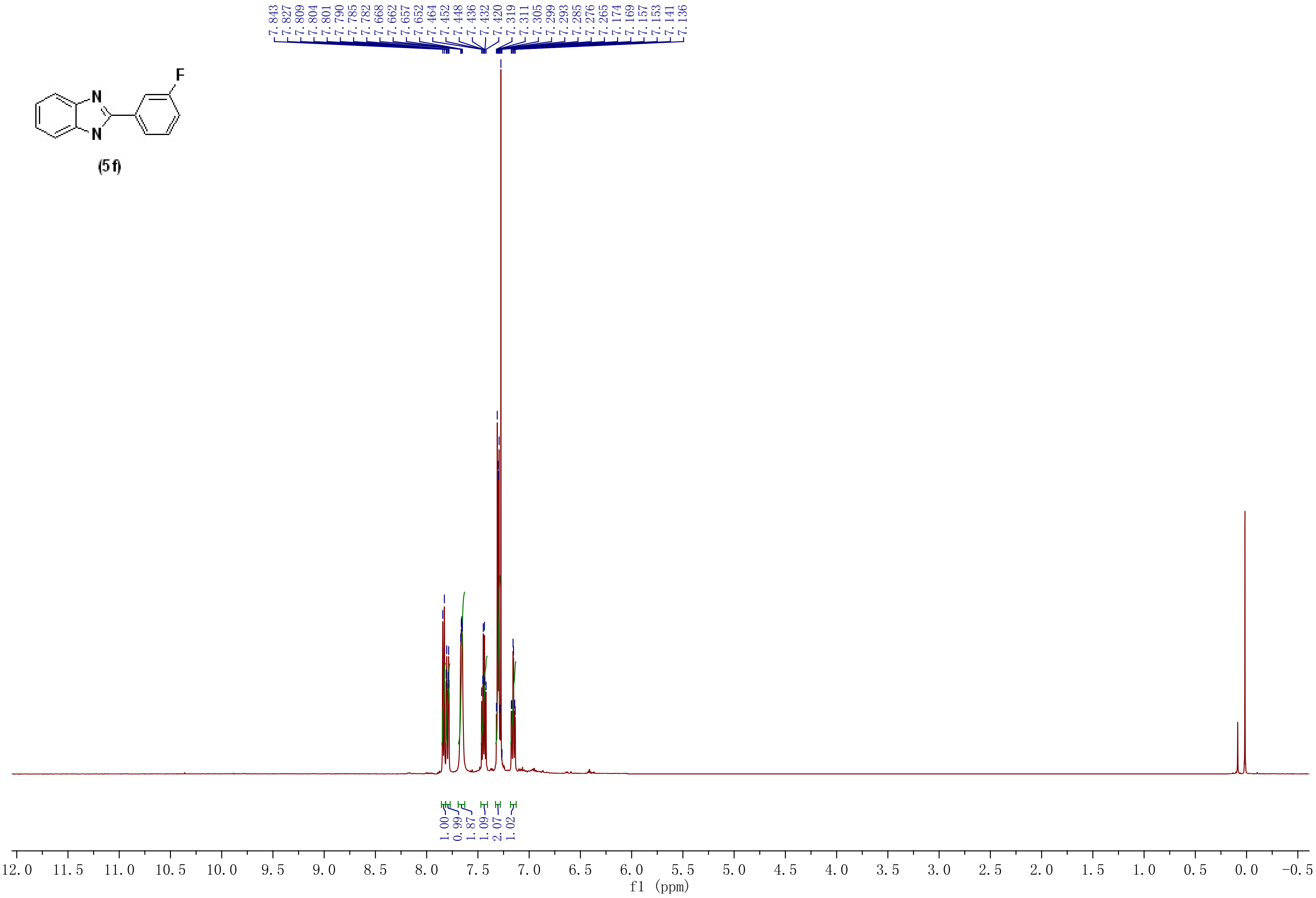

7.843
7.827
7.809
7.804
7.801
7.790
7.785
7.782
7.668
7.662
7.657
7.652
7.464
7.452
7.448
7.436
7.432
7.420
7.319
7.311
7.305
7.299
7.293
7.285
7.276
7.265
7.174
7.169
7.157
7.153
7.141
7.136
F
N
N
(5f)
1.00
0.99
1.87
1.09
2.07
1.02
12.0 11.5 11.0 10.5 10.0 9.5 9.0 8.5 8.0 7.5 7.0 6.5 6.0 5.5 5.0 4.5 4.0 3.5 3.0 2.5 2.0 1.5 1.0 0.5 0.0 -0.5
f1 (ppm)

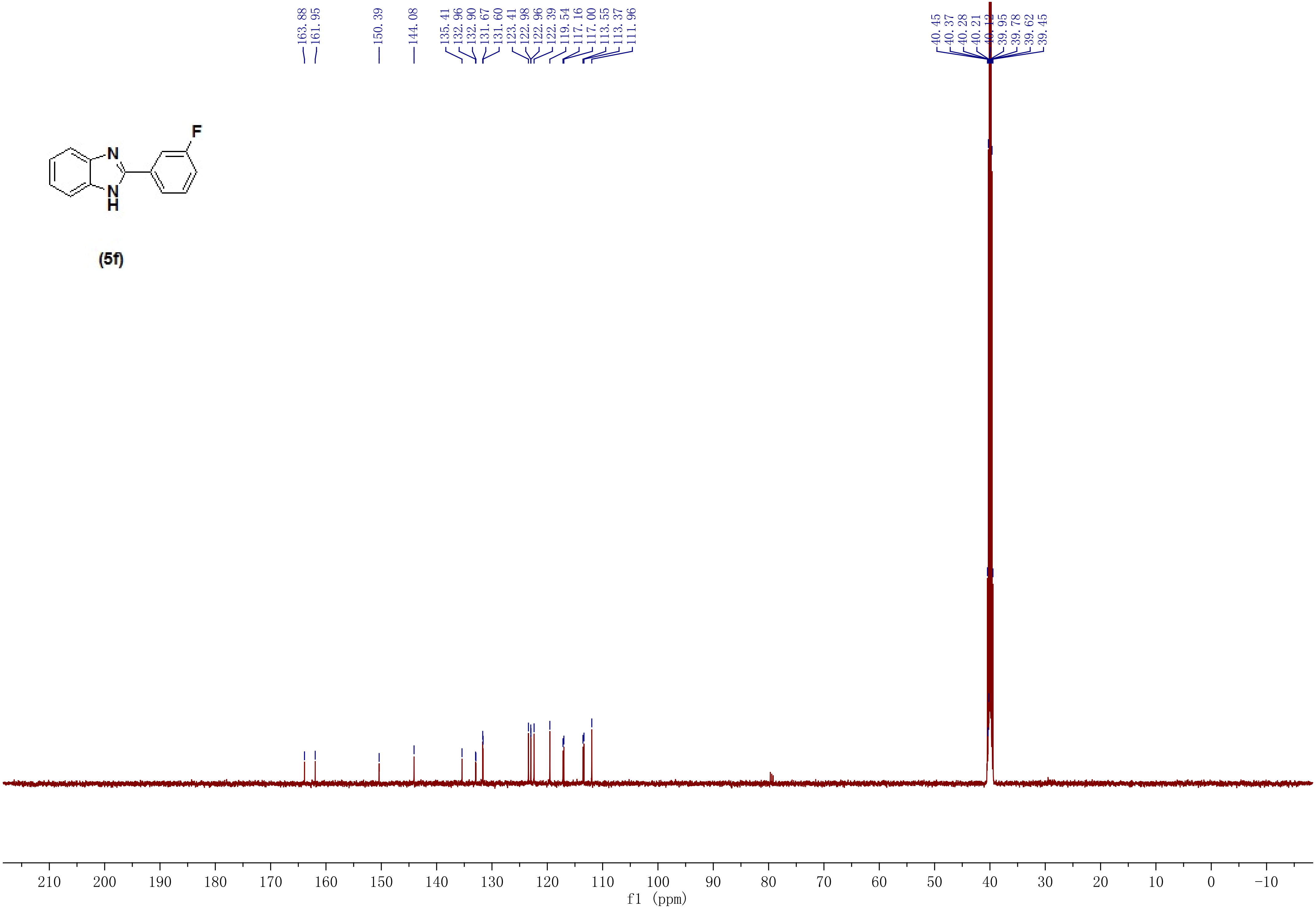
163.88
161.95
150.39
144.08
135.41
132.96
132.90
131.67
131.60
123.41
122.98
122.96
122.39
119.54
117.16
117.00
113.55
113.37
111.96
40.45
40.37
40.28
40.21
40.12
39.95
39.78
39.62
39.45
F
N
N
H
210 200 190 180 170 160 150 140 130 120 110 100 90 80 70 60 50 40 30 20 10 0 −10
f1 (ppm)

(5f)
